# Adipose Stromal Cells Amplify Angiogenic Signaling *via* the VEGF/mTOR/Akt Pathway in a Murine Hindlimb Ischemia Model: A *3D* Multimodality Imaging Study

**DOI:** 10.1371/journal.pone.0045621

**Published:** 2012-09-20

**Authors:** Weiwei Fan, Dongdong Sun, Junting Liu, Dong Liang, Yabin Wang, Kazim H. Narsinh, Yong Li, Xing Qin, Jimin Liang, Jie Tian, Feng Cao

**Affiliations:** 1 Department of Cardiology & Molecular Imaging Program, Xijing Hospital, Fourth Military Medical University, Xi’an, Shaanxi, China; 2 Life Sciences Research Center, School of Life Sciences and Technology, Xidian University, Xi’an, Shaanxi, China; 3 Department of Radiology & Institute for Stem Cell Biology and Regenerative Medicine, Stanford University School of Medicine, Stanford, California, United States of America; 4 Department of Plastic Surgery, Xijing Hospital, Fourth Military Medical University, Xi’an, Shaanxi, China; Medical University Innsbruck, Austria

## Abstract

Although adipose-derived stromal cell (ADSC) transplantation has been demonstrated as a promising therapeutic strategy for peripheral arterial disease (PAD), the mechanism of action behind the observed therapeutic efficacy of ADSCs remains unclear. This study was designed to investigate the long-term outcome and therapeutic behavior of engrafted ADSCs in a murine hindlimb ischemia model using multimodality molecular imaging approaches. ADSCs (1.0×10^7^) were isolated from Tg(*Fluc*-*egfp*) mice which constitutively express dual-reporter firefly luciferase and enhanced green fluorescent protein (Fluc^+^-eGFP^+^, mADSCs^Fluc+GFP+^), then intramuscularly injected into the hindlimb of BALB/c-nu mice after unilateral femoral artery ligation and excision. Abbreviated survival (∼5 weeks) of post-transplant mADSCs within the ischemic hindlimb was longitudinally monitored using noninvasive bioluminescence imaging (BLI), fluorescence imaging (FRI), and bioluminescence tomography with micro-computed tomography (BLT/micro-CT). Use of the BLT/micro-CT system enabled quantitative 3-dimensional (*3D*) imaging of the cells’ distribution and kinetics *in vivo*. Engrafted mADSCs improved blood perfusion recovery, ambulatory performance and prognosis of the ischemic hindlimb, probably by inducing angiogenesis and formation of collateral vessels, which could be visualized using laser Doppler perfusion imaging (LDPI), micro-CT angiography, vascular-cast imaging, and immunofluorescence. mADSCs augmented activation of the pro-angiogenic VEGF/mTOR/Akt pathway *in vivo*, even though the cells failed to incorporate into the host microvasculature as functional components. Downregulation of VEGF/mTOR/Akt signaling using small molecule inhibitors counteracted mADSC-induced angiogenesis and perfusion restoration. This study demonstrates for the first time the spatiotemporal kinetics and functional survival of transplanted mADSCs in a PAD model using *in vivo 3D* multimodality imaging. Our study indicates that mADSCs potentiate pro-angiogenic signal amplification *via* a VEGF/mTOR/Akt-dependent pathway, and thereby promote recovery from hindlimb ischemia.

## Introduction

Peripheral arterial disease (PAD) is a common condition affecting the circulation, in which the arteries that carry blood to the limbs become narrowed or clogged due to atherosclerosis. PAD also has a strong association with other life-threatening vascular diseases, such as coronary artery disease and carotid artery stenosis [1]. Unfortunately, it is likely that only a minority (∼5–35%) of PAD patients with critical limb ischemia (CLI) are suitable for surgical or percutanous revascularization therapy, while medications cannot effectively achieve a significantly improved prognosis [1], [2]. Cell-based transplantation provides a promising avenue for limb salvage from PAD [3]. Several lines of stem/progenitor and multipotent stromal cells have been employed to promote angiogenesis and restoration of peripheral perfusion, although some previous studies have yielded discrepant results [4]. The reasons contributing to outcome variation have not yet been fully elucidated. Primarily, little evidence has been provided to display the *in vivo* survival kinetics of transplanted cells, which further limits our understanding of the cells’ genuine behavior and therapeutic mechanism for tissue repair. Therefore, noninvasive approaches for tracking long-term functional survival of donor cells *in vivo* are definitely needed to better explain the heterogeneous results, as well as exploit more mechanism-driven cell-based therapeutic strategies.

Adipose-derived stromal cells (ADSCs) can be easily harvested, and have been proven to exert significant benefits for PAD models in the past decade [5]. Early clinical trials have also extended the application of ADSCs into PAD patients for improving blood perfusion recovery and ambulatory performance [5], [6]. However, neither the longitudinal survival of transplanted ADSCs nor the *in vivo* evidence of their therapeutic efficacy within the PAD model has been well established. More importantly, even though the beneficial effects of ADSCs have been confirmed *in vitro*, such as their multilineage differentiation and secretion of pro-angiogenic factors [7], their underlying mechanism of action *in vivo* remains unclear.

In the present study, we established murine ADSCs with stable expression of dual reporter genes (firefly luciferase and enhanced green fluorescent protein, Fluc^+^-eGFP^+^, mADSCs^Fluc+GFP+^), and employed multimodality molecular imaging strategies to visualize the functional survival of mADSCs^Fluc+GFP+^ in a murine CLI model. The tremendous growth of molecular imaging has allowed investigators to noninvasively obtain high-quality images that describe the fate of transplanted cells *in vivo* [8], [9]. Bioluminescence imaging (BLI), one of the most commonly used imaging modalities, has been preferred in our group’s previous noninvasive cell tracking studies due to its high sensitivity and low signal-to-noise ratio [10], [11], [12]. Nevertheless, two-dimensional (*2D*) BLI cannot depict the spatial distribution of cells inside the living body well [12]. Micro-computed tomography (micro-CT), however, has the advantage of high spatial resolution (10∼100 µm), excellent stereoscopy and short scan time. Accordingly, we have developed a dual-modality bioluminescence tomography(BLT)/micro-CT system to acquire quantitative, high-resolution and three-dimensional (*3D*) anatomical images for cell tracking [12], [13]. Hindlimb perfusion recovery was evaluated using *in vivo* laser Doppler perfusion imaging (LDPI), which matched collateral vessel remodeling well [14]. We attempted to 1) understand the longitudinal kinetics and outcome of engrafted mADSCs^Fluc+GFP+^ *in vivo* and 2) identify the therapeutic effect induced by mADSCs *in vivo* to provide insight into the involved mechanism(s).

## Materials and Methods

### Animals

Fluc^+^-eGFP^+^ transgenic mice [Tg(*Fluc*-*egfp*)] were bred on a C57BL/6a background to constitutively express Fluc-eGFP in all tissues and organs. Male syngenetic BALB/c nude mice (BALB/c-nu, *n* = 210, 8–10 week-old, 20–24 g, SPF) were used to construct the CLI model. All procedures were performed in accordance with the Guide for the Care and Use of Laboratory Animals by the National Institute of Health (USA). The protocol was approved by the Ethics Review Board of Fourth Military Medical University.

### Isolation and Identification of mADSCs

mADSCs^Fluc+GFP+^ were isolated from Tg(*Fluc*-*egfp*) mice. Cells were cultured and identified for immunophenotype and multipotency using previous procedures with minor modifications [15], [16] (see **Methods S1**).

### *In vitro* Reporter Gene Imaging and Assays

Dual-modality reporter gene imaging was performed to determine the Fluc-eGFP activity of mADSCs *in vitro* [10]. For BLI, mADSCs of different quantities were respectively suspended in 500 µl phosphate-buffered saline (PBS), incubated with the reporter probe D-luciferin (150 ng/µl, Invitrogen, USA), and sequentially imaged using a charge-coupled device (CCD, dual-modality) camera within Xenogen *In Vivo* Imaging System (IVIS, Caliper Life Sciences, USA), with the following parameters: binning: 4, F/Stop: 1, exposure time: 1 min. Peak BLI signal intensity was expressed in average radiance (photons/second/cm^2^/steridian, P·s^−1^·cm^−2^·sr^−1^) from a fixed-area region of interest (ROI). For fluorescence imaging (FRI), cell suspensions were directly imaged by CCD with its excitation wavelength at 465 nm/430 nm and emission filter at 560 nm. Fluorescence intensity was quantified by fluorescent calibrated units: average efficiency [ratio between radiance of the emission light (P·s^−1^) and excitation light (P·s^−1^)]. LivingImage 4.2 (Caliper) was used for imaging quantification. *In vitro* or e*x vivo* luciferase assays were performed on lysed cells or tissues *via* Luciferase Assay Buffer II (Promega, USA), using Dual-Luciferase Assay System (Promega). Luciferase activity was expressed in relative light unit (RLU) per mg protein. PBS without mADSC was used as control.

### CLI Model and Cell Delivery

BALB/c-nu mice (*n* = 210) were randomized into 3 groups (70 each matched for weight): 1) Sham+PBS (Sham); 2) CLI+PBS (Control); 3) CLI+mADSCs (ADSC). CLI was induced by ligating and excising the left femoral artery with all superficial and deep branches. Sham-operated mice received incision without artery ligation. Mice in the ADSC group were subjected to mADSCs^Fluc+GFP+^ (1.0×10^7^) delivery on post-operative day 1. Cells were suspended in 40 µl PBS and cautiously injected into the left gastrocnemius muscle using a 29-gauge insulin syringe (BD Biosciences, USA), while control and sham group animals received 40 µl PBS only.

### *In vivo* mADSCs^Fluc+GFP+^ Tracking *via* BLI/FRI/BLT/micro-CT

Noninvasive BLI, FRI, and BLT/micro-CT were performed to track the mADSCs^Fluc+GFP+^ *in vivo*. The chest fur of Tg(*Fluc*-*egfp*) mice was all shaved right before imaging to avoid inadvertent error in optical signal acquisition. For FRI, mice were anesthetized and directly imaged by CCD with its excitation wavelength at 465 nm/430 nm and emission filter at 560 nm. Fluorescence intensity was quantified by average efficiency using LivingImage 4.2. For BLI/BLT/micro-CT, mice were anesthetized and intraperitoneally injected with 150 mg/kg D-luciferin. BLI was conducted at 3-minute intervals until the peak signal was observed [10]. Photons emitted from a fixed-area ROI over the left hindlimb were quantified by average radiance (P·s^−1^·cm^−2^·sr^−1^). The spatial distribution and kinetics of mADSCs^Fluc+GFP+^ were visualized by our BLT/micro-CT system as previously described [12], [13]. The BLT spatial resolution can reach ∼1 mm and 80 µm for functional and structural imaging, respectively [12], [13]. Mice were placed onto a 37°C stage inside the light-tight chamber and scanned by CCD to get four-directional overlay images (binning: 4×4, exposure time: 3 min). The micro-CT imaging was performed using an X-ray tube with 15-*µ*m focal spot size, accompanied by a CCD X-ray detector incorporating a 4008×2672 pixel photodiode array with 9-*µ*m pixel pitch. The cone-beam reconstruction algorithm based on adaptive *hp*-finite element method (*hp*-FEM) was adopted for the quantification of cell tracking. BLT was quantified in units of total power (nanoWatt, nW) inside the living mouse. The same mouse was longitudinally scanned at 3-day intervals for a 6-week period by a blinded investigator.

### Serial Hindlimb Perfusion Imaging and Functional Assessment

To serially monitor the blood perfusion recovery of the ischemic hindlimb, mice were placed on a 37.4–38.0°C heating pad to minimize temperature variation, and then imaged using an LDPI analyzer (PeriScan-PIM3 Perimed AB, Sweden). The blood flux was quantified using perfusion ratio [PR, i.e. ratio of average LDPI index of ischemic to nonischemic(contralateral, self-control) hindlimb] by LDPIwin3.1.3 (Perimed AB). A cohort of mice in the ADSC and control group were subjected to LDPI after receiving intramuscular injection of vascular endothelial growth factor (VEGF) neutralizing monoclonal antibody (anti-VEGF mAb, n = 10, 20 µg·kg^–1^·day^–1^, R&D system, USA), PP242 (n = 10, 200 nM·kg^–1^·day^–1^, Sigma-Aldrich, USA) or triciribine (n = 10, 100 nM·kg^–1^·day^–1^, Sigma-Aldrich) from post-operative day 1 to day 35. Nonspecific isotype IgG or vehicle was administered as a control (n = 10, respectively). Semiquantitative functional assessments of the ischemic hindlimb were performed in a blinded manner using modified clinical scoring for ambulation and ischemic damage [17] (see **Methods S1**).

### *In vivo 3D* Visualization of Hindlimb Vasculature Remodeling

Gold nanoparticle (AuroVist-15 nm, Nanoprobes, USA) contrast-enhanced micro-CT imaging was employed to monitor formation of collateral vessels. Gold nanoparticles (40 mg/200 µl) were injected into the mouse *via* tail vein. After a 10-min contrast delay, mice were anesthetized and fixed on a computer-controlled electronic driving rotation stage. Micro-CT imaging was performed using 50-kVp X-ray tube voltage, with 1.4-mA tube current for 4 min through each hindlimb [12], [13]. To display *3D* architecture of hindlimb microvascular network, mice were sacrificed on day 21 and perfused with 10 ml casting polymer *via* the abdominal aorta for a vascular casting mould. The casted microvasculature was sputter-coated with gold and subsequently imaged using scanning electron microscopy (SEM, S-3400N, Hitachi, Japan).

### Histological Analysis of mADSC-induced Angiogenesis

Paraffin sections of the left gastrocnemius muscle tissue were obtained and sequentially analyzed using rat monoclonal anti-CD31 (1∶50, ab7388, Abcam, USA) immunohistochemistry staining to visualize CD31(PECAM-1)-positive vessels. Triple immunofluorescence staining using rat anti-CD31 (1∶100), FITC-conjugated goat anti-GFP (1∶200, ab6662, Abcam) and 4′,6-diamidino-2-phenylindole (DAPI) was performed on frozen sections of gastrocnemius muscle to show the relationship of mADSCs^Fluc+GFP+^ and CD31^+^ microvasculature (see **Methods S1**).

### Western Blot and ELISA

Gastrocnemius muscle tissue was harvested on day 0, day 3, and day 7 for Western blot and ELISA using our previously described protocols [18], [19] (see **Methods S1**).

### Statistics

Results are expressed as mean±standard deviation (SD). SPSS17.0 (SPSS Inc., USA) and Prism5.0 (GraphPad Software, USA) were used to perform the one-way analysis of variance (ANOVA) for evaluating the differences in bioluminescence radiance, total power, fluorescence efficiency, LDPI index (PR), semi-quantitative scores, vascular density, cell number, IOD and cytokines concentration, among the different experimental groups and different time points within each group. Pairwise multiple comparisons were to identify the parameters differences between the two groups using Tukey's test in conjunction with ANOVA. Data expressed as a proportion was assessed by Chi-square testing. A two-tailed *P*-value <0.05 was considered significant. Polynomial regression analysis was performed to evaluate the correlation between cell number and optical radiance *in vitro*.

## Results

### Characterization of mADSCs^Fluc+GFP+^

**Figure 1:**
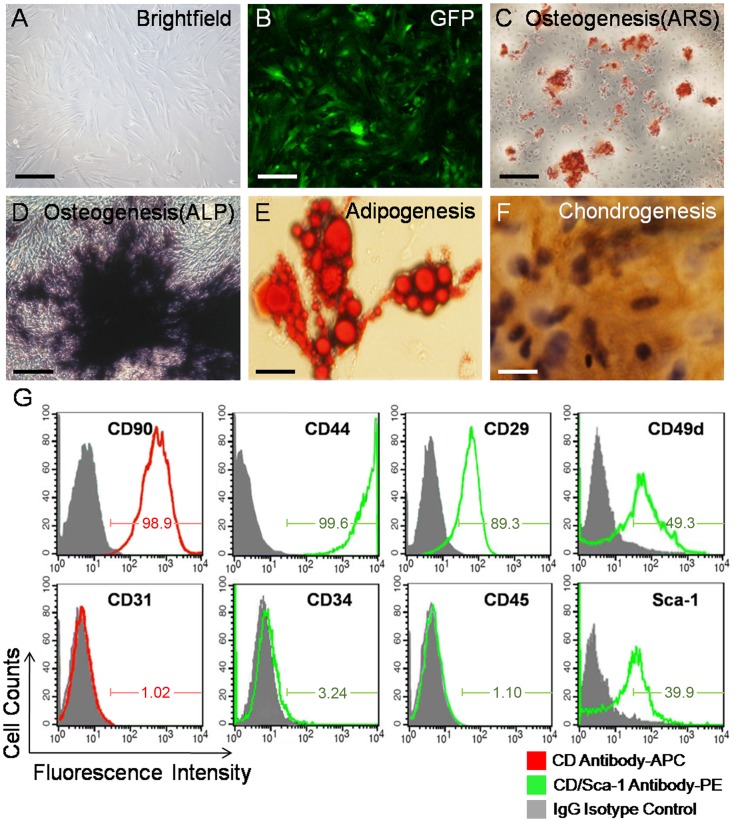
mADSCs^Fluc+GFP+^ characterization. Fibroblastoid morphology (**a**) and enhanced green fluorescent protein (eGFP) expression (**b**) of Fluc^+^−GFP^+^ murine adipose-derived stromal cells (mADSCs^Fluc+GFP+^, 3^rd^ passage). Multipotent differentiation of mADSC^Fluc+GFP+^ is shown in **c–f**. Osteogenesis was demonstrated using alizarin red-S (ARS, **c**) or alkaline phosphatase (ALP, **d**) staining. Adipogenesis and chondrogenesis were detected using oil red-O staining (**e**) and collagen-II immunohistochemistry staining (**f**) respectively. Flow cytometry of mADSCs labeled with phycoerythrin(PE)/allophycocyanin(APC)-conjugated cell markers or isotype IgG controls for immunophenotype identification (**g**). Scale bar represents 100 µm (a,b,c,d), 15 µm (e,f).

Optical imaging revealed that Tg(*Fluc*-*egfp*) mice persistently expressed dual Fluc-eGFP reporter genes in all tissues and organs (**Figure S1**). mADSCs^Fluc+GFP+^ could be abundantly isolated from adipose samples (∼10^6^ cells per gram raw tissue) and exhibited a distinctive *in vitro* fibroblastoid morphology, as mononuclear cells with a fusiform shape (**Figure 1a**). All the cells were GFP-positive (**Figure 1b**). mADSCs^Fluc+GFP+^ retained “stemness” properties. Following osteogenic induction, mineralized matrix deposition and alkaline phosphatase (ALP) activity were observed within mADSCs^Fluc+GFP+^ using alizarin red-S (**Figure 1c**) and ALP staining (**Figure 1d**), respectively. By oil Red-O (**Figure 1e**) and collagen-II immunohistochemistry staining (**Figure 1f**), adipogenic and chondrogenic capacity of mADSCs^Fluc+GFP+^ were also confirmed. Flow cytometry showed that most mADSCs^Fluc+GFP+^ expressed typical mesenchymal stem cell markers CD90 (98.9%), CD44 (99.6%) and CD29 (89.3%). mADSCs^Fluc+GFP+^ exhibited moderate expression of CD49d (49.3%) and stem cell marker Sca-1 (39.9%), and low expression of hematopoietic marker CD45 (1.10%), CD34 (3.24%) and endothelial marker CD31 (1.02%, **Figure 1g**).

### Quantification of mADSCs^Fluc+GFP+^ using Reporter Gene Imaging

**Figure 2:**
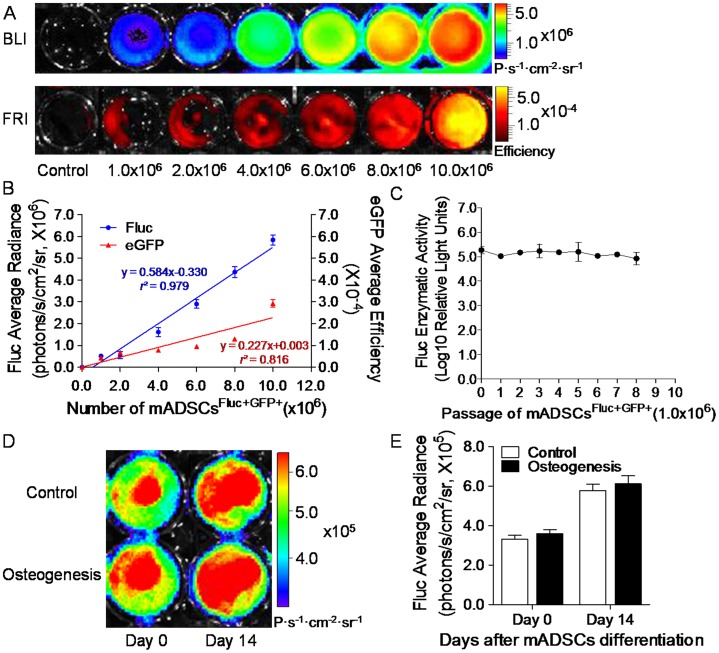
mADSCs^Fluc+GFP+^ quantification by reporter gene imaging. Representative bioluminescence/fluorescence images of mADSCs^Fluc+GFP+^ *in vitro* (**a**). Linear correlation of cell quantities with optical signal of firefly luciferase (Fluc) and eGFP is displayed, with correlation coefficient *r* ^2^ values and linear functions (**b**). The Fluc enzymatic activity of mADSC^Fluc+GFP+^ was stable in 8 passages (**c**). BLI (**d**) and quantification (**e**) of mADSCs^Fluc+GFP+^ over osteogenesis induction or normal culture (control) for 14 days. Colored scale bars represent Fluc bioluminescence intensity in photons/second/cm^2^/steridian (P·s^−1^·cm^−2^·sr^−1^), and eGFP fluorescence intensity in efficiency. n = 20 for each. Error bars: mean±SD.

mADSCs^Fluc+GFP+^ could emit dual-modality BLI/FRI signals (**Figure 2a**). BLI/FRI quantification exhibited robust linear correlation of cell number with Fluc average radiance (*r* ^2^ = 0.979), and eGFP average efficiency (*r* ^2^ = 0.816, **Figure 2b**). Importantly, mADSCs^Fluc+GFP+^ exhibited stable Fluc expression following subculture or multilineage differentiation. Fluc enzymatic assays showed no statistical difference among 8 passages of mADSCs^Fluc+GFP+^ (**Figure 2c**). After osteogenic induction, the mADSCs^Fluc+GFP+^ samples possessed an osteogenic cell phenotype, but maintained a BLI signal comparable to undifferentiated cells cultured in normal medium (*P*>0.05; **Figure 2d and 2e**). Following cell transplantation, *in vivo* BLI revealed the peak signal of mADSC^Fluc+GFP+^ appeared ∼15 min after D-luciferin administration (**Figure S2a** and **S2b**). Thus, the BLI signal obtained after 15 min was reported as a final value in all subsequent *in vivo* imaging studies.

### Transplanted mADSCs^Fluc+GFP+^ had Finite Survival in Ischemic Hindlimb

**Figure 3:**
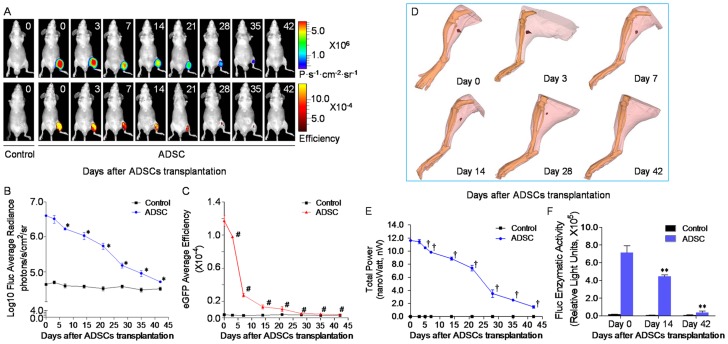
Longitudinal tracking of transplanted mADSCs^Fluc+GFP+^ using multimodality imaging *in vivo*. *In vivo* BLI/FRI/BLT/micro-CT tracked mADSC^Fluc+GFP+^ survival in the ischemic hindlimb of the same representative animal. Progressive decay of bioluminescence (**a** upper and **b**) and fluorescence signal intensity (**a** lower and **c**) over time is indicative of donor mADSCs^Fluc+GFP+^ death. Tomography and reconstruction of bioluminescence signals were performed by our BLT/micro-CT platform to show the *in vivo* kinetics of mADSCs^Fluc+GFP+^ (red mass) in detailed *3D* resolution (**d**). Reconstruction of bioluminescence total power (nanoWatts) also displayed a downward trend (**e**), confirmed by *ex vivo* Fluc assays (**f**). n = 20 for each. Error bars: mean±SD. *P<0.01 *vs.* day 0, #*P*<0.001 *vs.* day 0, †*P*<0.05 *vs.* day 0, **P<0.001 *vs.* day 0 (within ADSC group).

Multimodality BLI/FRI/BLT/micro-CT imaging facilitated the noninvasive tracking of transplanted mADSCs^Fluc+GFP+^ *in vivo*. After initial cell transplantation, BLI revealed a progressive decay of Fluc signal in the following 6 weeks (**Figure 3a**), from (4.02×10^6^±2.36×10^5^) P s^−1^ cm^−2^ sr^−1^ on day 0 to (1.08×10^6^±2.48×10^5^) P·s^−1^·cm^−2^·sr^−1^ on day 14, to background levels on day 42 (**Figure 3b**). FRI confirmed this trend with eGFP signal decay (**Figure 3a and 3c**). These findings indicated that the majority of transplanted mADSCs^Fluc+GFP+^ experienced progressive cell death in the ischemic hindlimb.

BLT/micro-CT further defined the *in vivo* spatiotemporal kinetics of mADSCs^Fluc+GFP+^. The tomography and *3D*-mode reconstruction of bioluminescence signals allowed noninvasive identification of transplanted mADSCs^Fluc+GFP+^ in detailed *3D* anatomic resolution (**Figure 3d** and **Movie S1, S2, S3, S4**). Furthermore, we have previously demonstrated that *3D*-reconstructed total power of bioluminescence signal displayed better linear correlation with the living cells number than *2D* BLI signal intensity *in vivo* [12]. BLT/micro-CT also detected the decreased total power of mADSCs^Fluc+GFP+^ compared with baseline (1.48±0.21 nW on day 42 *vs.* 11.6±0.16 nW on day 1, *P*<0.001, **Figure 3e**), confirmed by *ex vivo* Fluc assays (**Figure 3f**). More importantly, BLI/FRI/BLT/micro-CT specifically determined the retention of mADSCs^Fluc+GFP+^ at the graft site, as no incremental optical signal was detected in other locations of the hindlimb or whole body.

### Transplanted mADSCs Improved Blood Perfusion and Ambulatory Function

**Figure 4:**
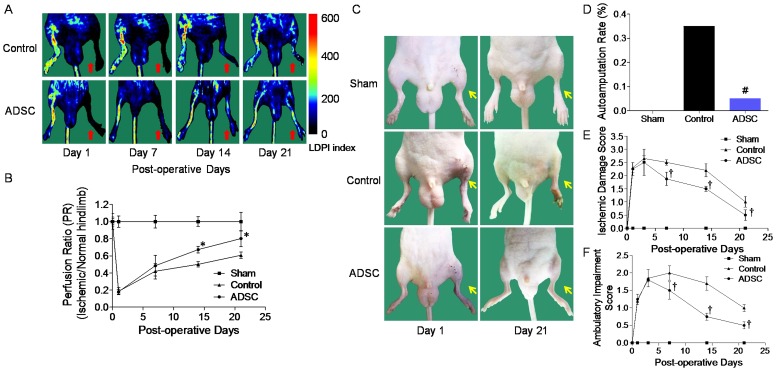
Blood perfusion imaging and function assessment of ischemic hindlimb. Laser Doppler perfusion imaging (LDPI) visualized dynamic changes in hindlimb perfusion (**a**). Colored scale bar represents blood flow velocity in LDPI index. Blood perfusion was quantified using perfusion ratio (PR), i.e., the ratio of average LDPI index of ischemic (red arrows) to nonischemic hindlimb (**b**). Representative photos show hindlimb autoamputation or salvage (yellow arrows) among groups on day 21 (**c**). Significantly lower autoamputation percentage (**d**), scores of ischemic damage (**e**) and ambulatory impairment (**f**) were observed in the ADSC group compared to the control group. n = 20 for each. Error bars: mean±SD. *P<0.001 *vs.* Control, #*P*<0.05 *vs.* Control, †*P*<0.001 *vs.* Control.

LDPI spatiotemporally visualized changes in peripheral blood perfusion (**Figure 4a**). On post-operative day 1, LDPI revealed a significantly lower level of perfusion in the operated hindlimb compared with the contralateral control hindlimb in all groups, suggesting effective CLI induction (**Figure 4b**). Although mice in the control group displayed a compensatory recovery of perfusion, transplanted mADSCs remarkably promoted restoration of blood perfusion, as the perfusion ratio in the ADSC group was significantly greater than the control group on day 14 and day 21 (day 14: 0.673±0.039 *vs.* 0.501±0.031, *P*<0.001). Transplanted mADSCs further improved the function and prognosis of the ischemic hindlimb. The autoamputation rate was significantly lower in the ADSC group than that in the control group on day 21 (5% *vs.* 35%, *P*<0.05, **Figure 4c and 4d**). Mice in the ADSC group, when compared with the control group, also had significantly lower ischemic damage scores (*P*<0.001, **Figure 4e**), and ambulatory impairment scores (*P*<0.001, **Figure 4f**).

### mADSCs Augmented Collateral Vessel Formation and Angiogenesis *in vivo*

**Figure 5:**
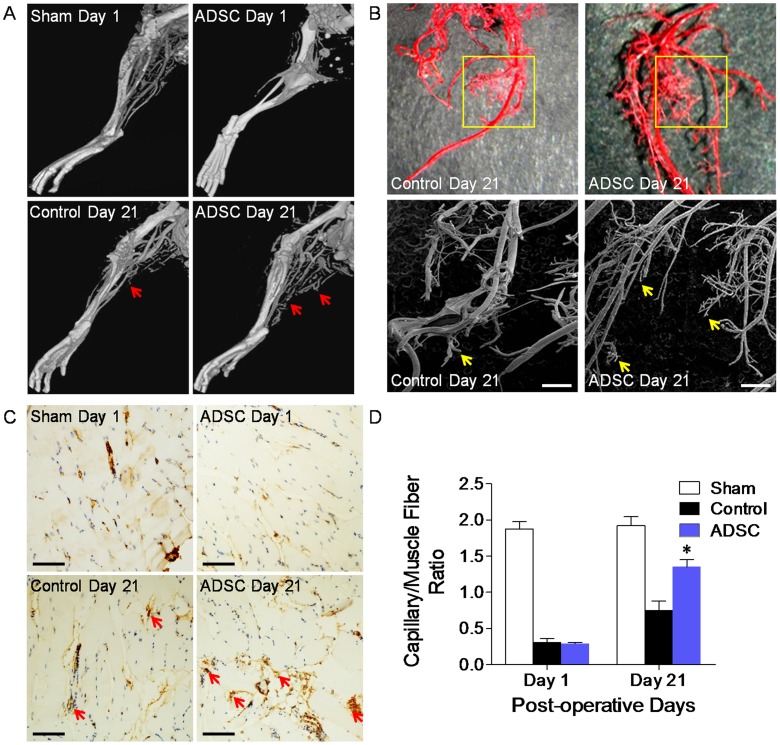
Transplanted mADSCs promoted collaterals formation and angiogenesis *in vivo*. Micro-CT angiography showed that mADSCs significantly increased collateral vessels formation (red arrows) on day 21 (**a**). Vascular cast (upper panels) and scanning electron microscopy (lower panels) visualized *3D* architecture of microvasculature and demonstrated the enhanced angiogenesis (yellow squares and arrows) in ADSC-treated animals compared to control group animals on day 21 (**b**). Immunohistochemical staining (**c**) and quantitative analysis (**d**) displayed higher CD31-positive capillary (red arrows) to muscle fiber ratio in the ADSC group compared to the control group on day 21. n = 20 random fields. Scale bar represents 10 µm (b), 100 µm (c). Error bars: mean±SD. *P<0.01 *vs.* Control.

Contrast-enhanced micro-CT angiography and corresponding *3D*-mode reconstruction demonstrated that mADSCs significantly increased hindlimb collateral formation *in vivo* when compared with control animals (**Figure 5a**). Furthermore, our vascular casting model with SEM imaging clearly depicted the *3D* architecture of the vascular network and determined that mADSCs promoted microvasculature formation, as the capillary density was significantly higher in animals receiving ADSCs rather than PBS on day 21 after injection (**Figure 5b**). Immunohistochemistry further validated that the CD31^+^ capillary to muscle fiber ratio of the ischemic hindlimb was significantly higher in mice receiving mADSCs rather than PBS on day 21 (1.35±0.10 *vs.* 0.75±0.13, *P*<0.01), although the ratio was comparable on day 1 (**Figure 5c and 5d**). These findings demonstrated that the enhanced restoration of hindlimb ischemia in the ADSC group probably resulted from mADSC-induced angiogenesis and collateral vessel formation.

### mADSCs^Fluc+GFP+^ did not Incorporate into the Host Capillary Network

**Figure 6:**
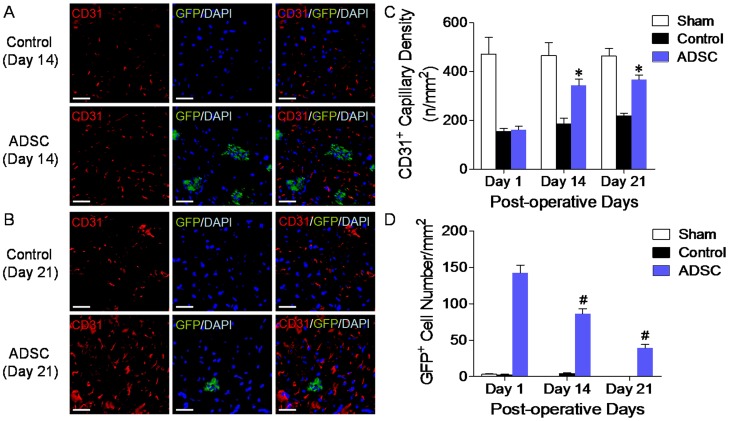
Immunofluorescence assessment of mADSC engraftment and angiogenesis. Confocal microscopy of ischemic gastrocnemius muscle tissue sections triply stained with GFP (green), endothelial marker CD31 (PECAM-1, red) and 4′,6-diamidino-2-phenylindole (DAPI, blue) visualized the para-vascular survival of mADSCs^Fluc+GFP+^ (**a** and **b**). Quantitative analysis of CD31^+^ blood vessels (**c**) and GFP^+^ cells (**d**) within the same-sized regions. n = 20 random fields. Scale bars represent 50 µm. Error bars: mean±SD. *P<0.001 *vs.* Control, #*P*<0.001 *vs.* Day 1 within ADSC group.

To further understand the link between donor mADSCs and amplified angiogenesis, we tracked the outcome of post-transplant mADSCs using immunofluorescence analysis, relying on mADSCs’ stable eGFP expression. Consistent with BLI/FRI/BLT/micro-CT findings, laser confocal microscopy revealed the retention of mADSCs^Fluc+GFP+^ clusters within native ischemic muscle (**Figure 6a and 6b**), suggesting successful engraftment and survival of the injected cells. More CD31^+^ blood vessels were found in the mADSC-treated ischemic region than the same-sized PBS-treated region (day 14: 342±26 *vs.* 185±23, n/mm^2^, *P*<0.001, **Figure 6c**). Importantly, mADSCs were found adjacent to the host microvasculature rather than incorporated into it. No engrafted mADSCs^Fluc+GFP+^ expressed CD31 *in vivo*, indicating little chance for the injected cells to become differentiated or incorporated into endothelial cells. Again, fewer mADSCs^Fluc+GFP+^ survived on day 21 than day 14 (**Figure 6d**). On day 35, very few GFP^+^ cells could be observed within the ischemic muscle, consistent with acute death of donor mADSCs *in vivo* (**Figure S3a**). Combined anti-VEGF mAb, PP242 (an ATP-competitive inhibitor of mammalian target of rapamycin, mTOR), and selective Akt/PKB inhibitor triciribine treatment with mADSCs counteracted mADSC-induced angiogenesis (**Figure S3a** and **S3b**).

### Transplanted mADSCs Amplified Angiogenic Signal *via* VEGF/mTOR/Akt Pathway

**Figure 7:**
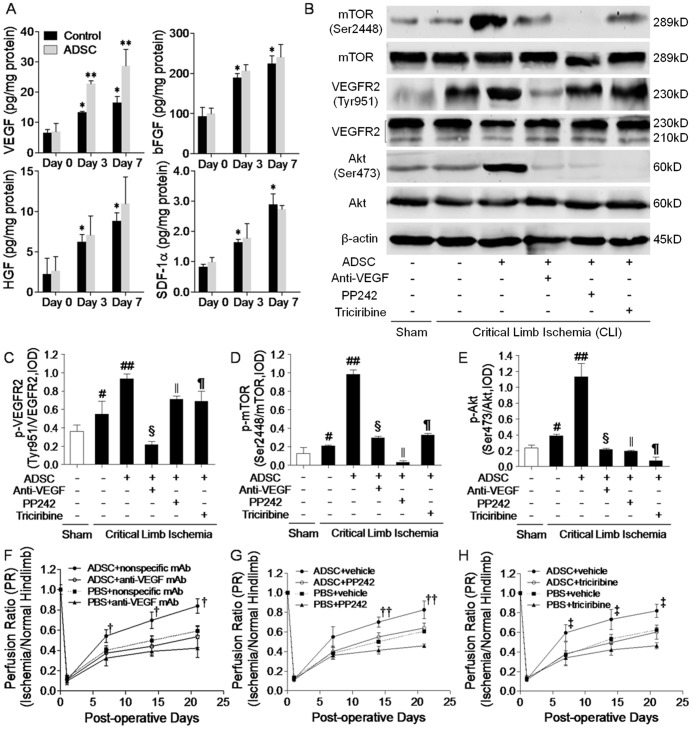
Transplanted mADSCs activated VEGF/mTOR/Akt pathway *in vivo*. Levels of vascular endothelial growth factor (VEGF), basic fibroblast growth factor (bFGF), hepatocyte growth factor (HGF) and stromal cell derived factor-1α (SDF-1α) in PBS or ADSC-treated ischemic hindlimbs on day 0 (baseline), 3 and 7 using ELISA (**a**). Western blot analysis (**b**) of VEGFR2(Tyr951)/VEGFR2 (**c**), mTOR(Ser2448)/mTOR (**d**), Akt(Ser473)/Akt (**e**) and β-actin expression within ischemic hindlimbs on day 7. Protein expression was quantified by the integrated optical density (IOD) ratio of each pair. n = 20 for each. LDPI follow-up showed that combined treatment of anti-VEGF monoclonal antibody (mAb) (**f**), PP242 (**g**) or triciribine (**h**) with mADSCs attenuated mADSC-induced blood perfusion restoration, compared with combined treatment of nonspecific IgG or vehicle controls. Error bars: mean±SD. *P<0.05 *vs.* day 0 within Control group, **P<0.05 *vs.* Control, #*P*<0.05 *vs.* Sham, ##*P*<0.001 *vs.* Control, §*P*<0.001 *vs.* ADSC, ‖*P*<0.01 *vs.* ADSC, ¶*P*<0.01 *vs.* ADSC, †*P*<0.01 *vs.* ADSC+anti-VEGF mAb, ††*P*<0.001 *vs.* ADSC+PP242, ‡*P*<0.05 *vs.* ADSC+triciribine.

The transience of transplanted mADSCs indicated that mADSCs likely activated angiogenic signaling to promote neovasculogenesis, rather than functionally integrated into the host vasculature. In the present study, several putative mADSC-secreted angiogenic factors were detected in the ischemic hindlimb using *ex vivo* ELISA, and amongst them only VEGF experienced a remarkable elevation in the group receiving ADSCs compared with the control group on day 3 and day 7 (day 7: 28.6±5.5 *vs.* 16.5±2.0, pg/mg protein, *P*<0.05, **Figure 7a**). Although basic fibroblast growth factor (bFGF), hepatocyte growth factor (HGF) and stromal cell derived factor-1α (SDF-1α) have been previously established as members of the ADSC’s secretome [20], no significant difference in their levels was observed in the ischemic hindlimb between the ADSC and control group (*P>*0.05, respectively). Intriguingly, hosts exhibited a spontaneous upregulation of VEGF, bFGF, HGF and SDF-1α on day 3 and day 7 when compared to day 0 (*P<*0.05 within control group, respectively), indicating some degree of endogenous angiogenic factors mobilization in response to hindlimb ischemia.

Western blot results revealed an activation of the VEGFR2/mTOR/Akt pathway within the ischemic hindlimb in the control group (**Figure 7b**). Engrafted mADSCs significantly promoted activation of VEGFR2 (KDR/Flk-1, **Figure 7c**), which acts as the major receptor for VEGF-induced angiogenesis [21]. mADSCs further upregulated the phosphorylation of mTOR (**Figure 7d**) and Akt (**Figure 7e**), known as downstream targets of VEGF/VEGFR2 [22], [23]. Anti-VEGF mAb treatment with mADSCs severely inhibited VEGFR2/mTOR/Akt activation, and abrogated mADSC-mediated restoration of perfusion as assessed by serial LDPI monitoring (**Figure 7f**). PP242 (**Figure 7g**) or triciribine (**Figure 7h**) treatment with mADSCs also downregulated activation of the VEGFR2/mTOR/Akt pathway and attenuated the restoration of perfusion induced by mADSCs. Anti-VEGF mAb, PP242 or triciribine also suppressed the spontaneous perfusion recovery in ischemic hindlimbs that had not been treated with mADSCs. Overall, activation of VEGF/VEGFR2/mTOR/Akt signaling at least partly contributed to post-ischemic and mADSC-mediated hindlimb angiogenesis.

## Discussion

Cell-based transplantation as an alternative PAD therapy would be of great significance, because PAD is associated with a high risk of amputation and subsequent mortality if surgical/endovascular revascularization fails or is unavailable [1]. Restoring peripheral perfusion is central to the success of PAD treatment [1], [2]. Stenosis of peripheral arteries can reduce blood and oxygen supply, resulting in limited physical capacity and increased risk of tissue loss [1]. A variety of stem/progenitor cells, such as bone marrow-derived endothelial progenitor cell (EPC) and mononuclear cell (BMMNC), have been identified for their pro-angiogenic potential to improve perfusion and ultimately allow limb salvage as an experimental or clinical option [3], [6]. Nonetheless, uncertainty regarding the cell engraftment rate, along with mixed therapeutic outcomes, seriously restricts the development of cell-based therapy. One key issue is our limited understanding of the *in vivo* kinetics and functional survival of transplanted cells. Molecular imaging provides feasible tools for the evaluation and translation of cell transplantation therapy [8], [9]. Our previous work has shown that BLI is an accurate and sensitive method for tracking cells *in vivo* with as few as 500 cells [8], [10], [11]. Using BLI, two recent studies revealed that engrafted BMMNCs were short-lived and failed to yield any significant benefit in murine myocardial infarction (MI) or PAD model [24], [25]. Accordingly, the changing face of the cell therapy field with a new emphasis on efficacy makes a thorough understanding of the long-term outcome of transplanted stem cells *in vivo* an imperative.

Recently, adipose tissue has emerged as another attractive source of regenerative cells, as ADSCs can promote postnatal vascular growth in ischemic models [7]. However, the fate and functional mechanism of transplanted ADSCs in a PAD model has not been well defined. In the present study, BLI was used to longitudinally track the progressive death of engrafted mADSCs^Fluc+GFP+^ in murine ischemic hindlimb. In BLI, photons emitted endogenously from the tissue are due to specific ATP-dependent luciferase-luciferin chemical reaction, which acts as a hallmark of living Fluc^+^ cells *in vivo*. Nevertheless, the information provided by *2D* BLI is limited by the overlapping anatomic structures of deep tissue [12]. Furthermore, a “reporter gene silencing” phenomenon due to the potential immunogenicity of eGFP [26], [27], may lead to eGFP downregulation *in vivo* and an earlier decay of eGFP signals following mADSCs^Fluc+GFP+^ transplantation. FRI also has technical limitations. As external illumination is required for inciting excitation light in FRI, photons emitted from endogenous particles are enhanced and the specific optical signal is unfavorably attenuated [12]. Therefore, we newly constructed a *3D* prototype BLT/micro-CT system for mADSCs^Fluc+GFP+^ tracking. Using BLT/micro-CT, multi-view BLI around the animal and tomographic reconstruction can recover the original signal, while the bioluminescence location can be registered into the *3D*-anatomic structure by micro-CT volume reconstruction [12], [13]. In this experiment, BLT/micro-CT provided detailed *3D* images for the cells’ location and kinetics, and energy reconstruction also determined the abbreviated lives of mADSCs^Fluc+GFP+^ *in vivo*.

Although the engrafted mADSCs were short-lived, they significantly benefited the ischemic hindlimb. Irrespective of spontaneous compensation, LDPI revealed that transplanted mADSCs overtly promoted the restoration of peripheral perfusion, probably resulting from mADSC-induced collateral vessel formation and angiogenesis. Micro-CT angiography demonstrated that mADSCs enhanced collateral formation *in vivo*, while vascular casting with SEM further depicted the morphology of mADSC-induced microvasculature remodeling, which was confirmed by histological analysis. mADSCs’ therapeutic effects also contributed to the functional recovery of ischemic hindlimb, as evidenced by improvement in motor function and reduction in autoamputation rate. Our observations are consistent with the findings of Miranville and colleagues [28]. We have further validated the mADSCs’ functional engraftment over time using comprehensive *in vivo* imaging studies.

To explore the mechanism of action behind mADSC-induced angiogenesis, we further examined the post-transplant behavior of mADSCs. Our data demonstrated that mADSCs probably did not experience proliferation or migration following engraftment, as no ectopic signal or signal enhancement at the graft site was detected using BLI/FRI/BLT/micro-CT. Previous studies have showed that ADSCs can adopt an endothelial or pericytic phenotype *in vitro*, suggesting the potential of ADSCs to stabilize endothelial networks *in vitro* and to engage in vascular formation *in vivo* [29], [30]. In the current PAD model, however, engrafted mADSCs failed to differentiate or directly incorporate into the native microvasculature network, even though the cells possessed multilineage potential *in vitro*. The para-vascular survival of mADSCs is more in accordance with their hypothetical paracrine action [31].

Several soluble factors, e.g. growth factors (VEGF, HGF, bFGF, etc.) and stromal factors (SDF-1, etc.) have been identified within ADSCs’ secretome [20]. In this experiment, engrafted mADSCs significantly activated VEGF expression in the ischemic hindlimb. Importantly, anti-VEGF mAb administration completely inhibited the pro-angiogenic effect of mADSCs. VEGF plays a pivotal role in angiogenesis not only through upregulating pro-angiogenic genes, but also through recruiting accessory cells close to angiogenic vessels [32]. We previously demonstrated that VEGF was capable of facilitating the survival and therapeutic function of engrafted stem cells in a MI model, while VEGF-induced nascent vasculature was being formed [18]. ADSCs can respond to a hypoxic stimulus by modulating VEGF expression, which facilitates their adaption to the hypoxic environment, shown in Rehman and coworkers’ study [31]. Accordingly, VEGF secretion is a protective response of ADSCs to ischemia. Apart from VEGF, no significant differences in HGF, bFGF and SDF-1α level was assessed between mADSCs and PBS-treated ischemic hindlimb, indicating some degree of limitation in mADSCs’ secretory function following transplantation [33].

Engrafted mADSCs activated VEGFR2, which can mediate mitogenic signaling to elicit angiogenic response and further, to functionally compensate for ischemia in the cardiovascular system [34], [35]. Defect in VEGF/VEGFR2 leads to severe impairment of physiologic angiogenesis following tissue ischemia [34], [35]. Furthermore, phosphorylation of mTOR/Akt, as key molecules within pro-angiogenic signaling [36], was upregulated by engrafted mADSCs. VEGF-induced mTOR activation has been demonstrated to be necessary for angiogenesis in tumor models, while inhibition of VEGF and mTOR works synergistically to kill cancer cells and prevent tumor angiogenesis [22]. Akt is another essential target in VEGF-mediated angiogenesis [23]. In this study, mTOR/Akt activation contributed considerably to mADSC-mediated angiogenesis, as antagonism of mTOR/Akt remarkably attenuated mADSCs’ pro-angiogenic function. Intriguingly, activated mTOR/Akt can further promote VEGF expression in a feedforward manner [22], [37]. Overall, a sequential VEGF/VEGFR2/mTOR/Akt activation loop is conceivable in PAD models, with transplanted mADSCs serving to amplify the loop to facilitate angiogenesis.

### Conclusions

The salient findings from our study represent the first attempt to longitudinally investigate the functional survival of transplanted ADSCs within a PAD model, using *in vivo 3D* multimodality molecular imaging. We demonstrated that 1) transplanted mADSCs were short-lived (∼5 weeks) in the ischemic hindlimb, while use of the noninvasive BLT/micro-CT enabled quantitative *3D* imaging for cells’ tracking *in vivo*; 2) engrafted mADSCs could promote the recovery of blood perfusion, ambulation and prognosis of the ischemic hindlimb through collateral vessel formation and angiogenesis, probably *via* a VEGF/mTOR/Akt-dependent pathway. These findings indicate that mADSCs transplantation hold potential for therapeutic angiogenesis in PAD. A comprehensive assessment of the engrafted cells’ behavior *in vivo* is a definite prerequisite before the cell is introduced as a clinical application. Although the multimodality imaging system can be applied as a powerful tool for *in vivo* cell monitoring in small animal models, the limited light penetration, the potential risk associated with genetically-modified cells, and the potential immunogenicity and toxicity of probe substrates [9], may prohibit further clinical investigation. Nonetheless, by optimizing the reporter gene induction and various imaging approaches, our *3D* multimodality imaging platform may yield valuable insight into long-term cell tracking and facilitate cell-based therapy evaluation *in vivo*.

## Supporting Information

Figure S1 Bioluminescence/fluorescence imaging (BLI/FRI) for Tg(*Fluc*-*egfp*) mice. Colored scale bars represent Fluc bioluminescence intensity in photons/second/cm^2^/steridian (P·s^−1^·cm^−2^·sr^−1^), and eGFP fluorescence intensity in efficiency.
(TIF)

Figure S2 Representative BLI of transplanted cells in the left ischemic hindlimb (a). *In vivo* BLI demonstrated that peak bioluminescence signal was obtained at ∼15 min after D-luciferin administration (b). Error bars: mean±SD.
(TIF)

Figure S3 Assessment of hindlimb angiogenesis following treatment of mADSCs with inhibitors of VEGF/mTOR/Akt pathway. Immunofluorescence assay (a) demonstrated that combined treatment of anti-VEGF monoclonal antibody (mAb), PP242 or triciribine with mADSCs abrogated mADSC-mediated angiogenesis (b). n = 20 random fields. Scale bars represent 50 µm. Error bars: mean±SD. †*P*<0.001 *vs.* Control, **P<0.001 *vs.* ADSC, ##*P*<0.001 *vs.* ADSC, ††*P*<0.001 *vs.* ADSC.
(TIF)

Movie S1 BLT/micro-CT *3D*-reconstructed image of mADSCs^Fluc+GFP+^ *in vivo* on post-transplant day 0.
(AVI)

Movie S2 BLT/micro-CT *3D*-reconstructed image of mADSCs^Fluc+GFP+^ *in vivo* on post-transplant day 3.
(AVI)

Movie S3 BLT/micro-CT *3D*-reconstructed image of mADSCs^Fluc+GFP+^ *in vivo* on post-transplant day 7.
(AVI)

Movie S4 BLT/micro-CT *3D*-reconstructed image of mADSCs^Fluc+GFP+^ *in vivo* on post-transplant day 14.
(AVI)

Methods S1 Document contains supplemental methods.
(DOCX)
