# Supplementary figures and images for: Adipose Stromal Cells Amplify Angiogenic Signaling via the VEGF/mTOR/Akt Pathway in a Murine Hindlimb Ischemia Model: A 3D Multimodality Imaging Study
Source: PLoS One. 2012 Sep 20;7(9):e45621. doi: 10.1371/journal.pone.0045621 (PMC3447795; doi:10.1371/journal.pone.0045621)

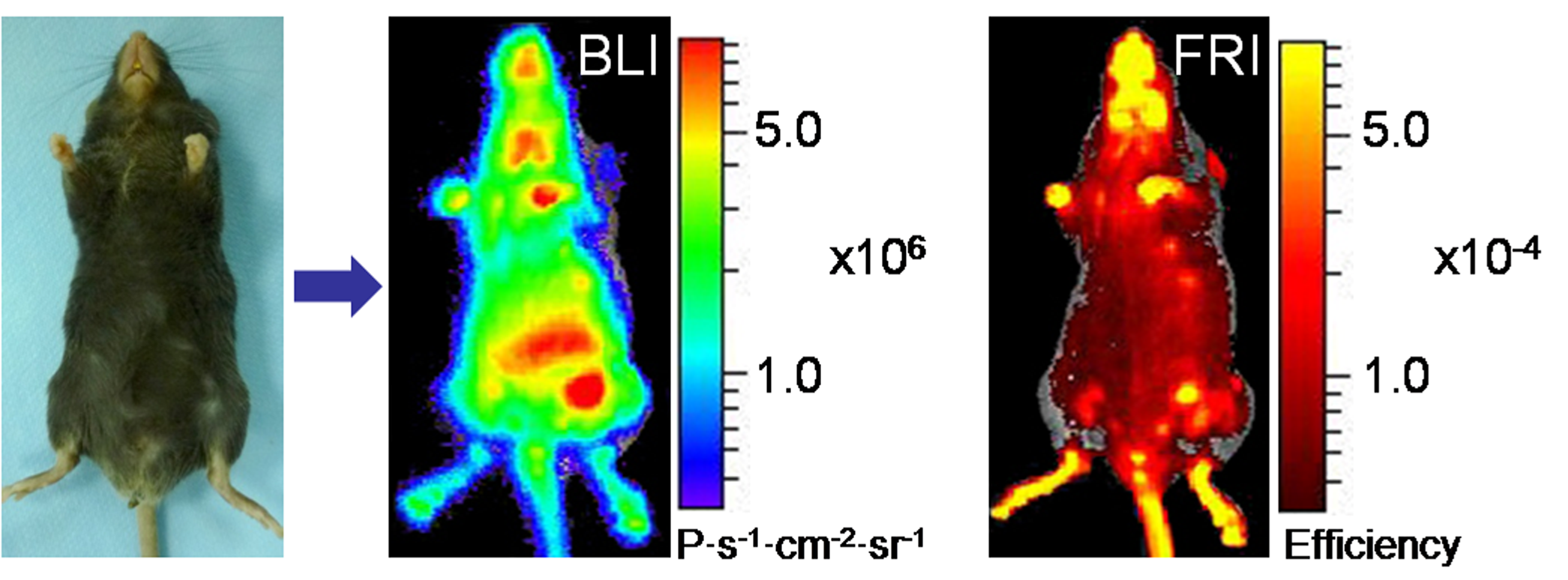

Supplement: Figure S1 — Bioluminescence/fluorescence imaging (BLI/FRI) for Tg(Fluc-egfp) mice. Colored scale bars represent Fluc bioluminescence intensity in photons/second/cm2/steridian (P·s−1·cm−2·sr−1), and eGFP fluorescence intensity in efficiency. (TIF) [file pone.0045621.s001.tif]

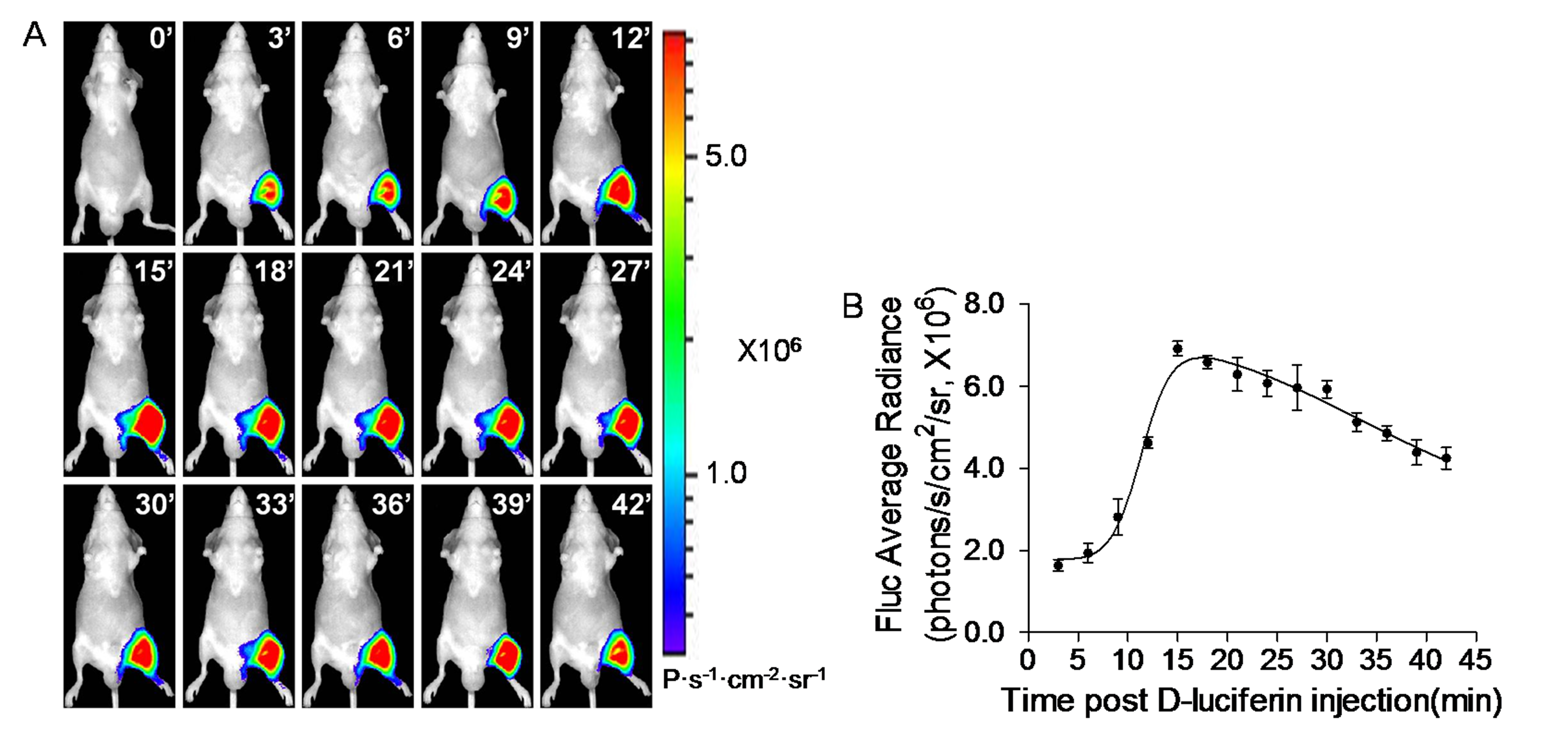

Supplement: Figure S2 — Representative BLI of transplanted cells in the left ischemic hindlimb (a). In vivo BLI demonstrated that peak bioluminescence signal was obtained at ∼15 min after D-luciferin administration (b). Error bars: mean±SD. (TIF) [file pone.0045621.s002.tif]

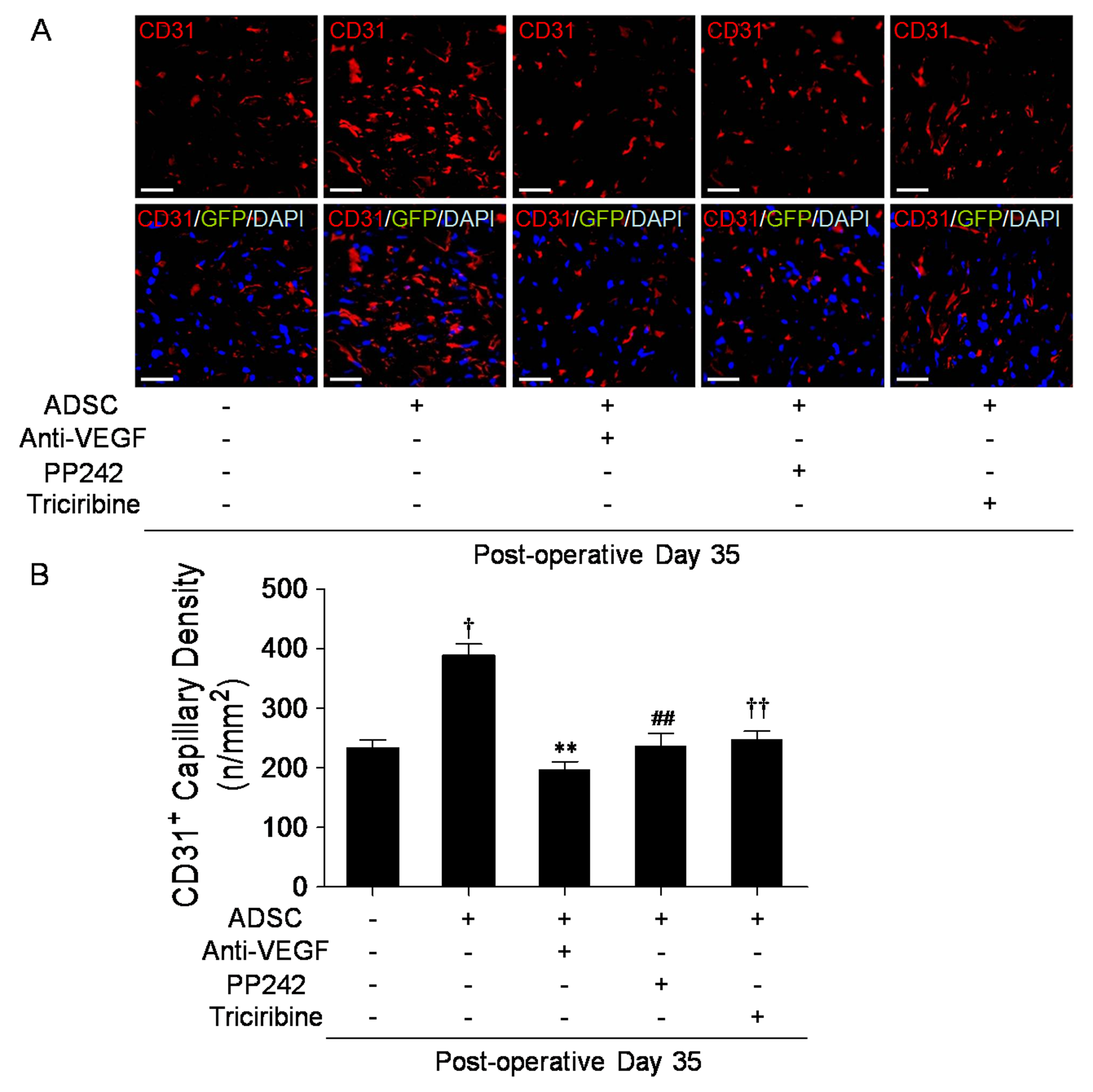

Supplement: Figure S3 — Assessment of hindlimb angiogenesis following treatment of mADSCs with inhibitors of VEGF/mTOR/Akt pathway. Immunofluorescence assay (a) demonstrated that combined treatment of anti-VEGF monoclonal antibody (mAb), PP242 or triciribine with mADSCs abrogated mADSC-mediated angiogenesis (b). n = 20 random fields. Scale bars represent 50 µm. Error bars: mean±SD. †P<0.001 vs. Control, **P<0.001 vs. ADSC, ##P<0.001 vs. ADSC, ††P<0.001 vs. ADSC. (TIF) [file pone.0045621.s003.tif]
