# Supplementary material for: Adipose Stromal Cells Amplify Angiogenic Signaling via the VEGF/mTOR/Akt Pathway in a Murine Hindlimb Ischemia Model: A 3D Multimodality Imaging Study
Source: PLoS One. 2012 Sep 20;7(9):e45621. doi: 10.1371/journal.pone.0045621 (PMC3447795; doi:10.1371/journal.pone.0045621)
Supplement: Methods S1 — Document contains supplemental methods. (DOCX) [file pone.0045621.s008.docx]

**Supplemental methods**

**Animal anesthesia**

All animal procedures were conducted in accordance with the Guide for the Care and Use of Laboratory Animals by the National Institute of Health [1] and approved by Fourth Military Medical University ethics review board. For *in vivo* studies, mice were anesthetized by 2% isoflurane in 0.3 L/min oxygen inhalation using Small Animal Anesthesia Machine-VMC (Matrx Medical Inc, USA) during the procedure. The adequacy of anesthesia was monitored by disappearance of pedal withdrawal reflex. For *ex vivo* studies, mice were sacrificed by euthanasia. The adequacy of sacrifice was monitored from the disappearance of heart beat and breath.

**Cell isolation and culture**

mADSCs were isolated and expanded using previous procedures with minor modifications [2,3,4]. Raw adipose tissues from Tg(*Fluc*-*egfp*) mice were washed thoroughly with sterile phosphate buffered saline (PBS) with 1% antibiotic/antimycotic to prevent contaminating blood cells and bacterium. Washed tissues were digested with 0.2% collagenase I (Invitrogen, USA) at 37°C for 60-75 min with gentle agitation to release the cellular fractions. Then the suspension was centrifuged at 200×*g* for 10 min. The cellular pellet was resuspended, filtered through a 100-μm mesh filter (Millipore, Germany) to remove debris and incubated in Dulbecco’s modified Eagle’s medium/F-12 (DMEM/F-12, Hyclone, USA) with 10% (vol/vol) fetal bovine serum (FBS, Hyclone) for 48 h at 37°C/5%CO_2_. Following incubation, the plates were washed extensively with PBS to remove residual nonadherent cells. Then the adherent cells were digested, plated (2×10^4^/cm^2^) in 75cm^2^ flask (Corning, USA) and cultured in DMEM/F-12/10%FBS supplemented with 5 ng/ml basic fibroblast growth factor (bFGF, Peprotech, USA) and 1% antibiotic/antimycotic. The expansion medium was refreshed every two days. When cells reached 80% confluence, they were detached by 0.05%trypsin + 0.02%EDTA (Invitrogen, USA), and then re-plated at a density of 5×10^4^/cm^2^ in cell culture flasks. The doubling time of mADSCs was measured by cell counting and was approximately 36 h. mADSCs could be abundantly isolated (~10^6^ cells per gram raw adipose tissue).

**Immunophenotypic analyses**

For immunophenotype identification [3,4], mADSCs (3^rd^ passage) were detached with 0.05%trypsin/0.02%EDTA (Invitrogen), and fixed for 30 min in ice-cold 1% paraformaldehyde. Cell aliquots (1.0×10^6^) were washed in flow cytometry buffer (FCB: 2% FBS and 0.2% Tween-20 in PBS) and incubated in FCB containing fluorescein phycoerythrin(PE)-conjugated monoclonal rabbit anti-mouse CD29, CD31, CD34, CD44, CD45, CD49d, CD90 and Sca-1 (BD Pharmingen, USA) for 30 min at 4°C. Cells were also stained with isotype-identical nonspecific IgG (BD Pharmingen) as control to assess background fluorescence. The stained cells, excluding dead ones, were analyzed by FACscan argon laser cytometer (BD Biosciences, USA). FlowJo 5.7 (Tree Star Inc., USA) was used for the following data analysis.

***In vitro* multilineage differentiation**

For osteogenic differentiation [4], mADSCs (3^rd^ passage) were induced by feeding them for 2.5 weeks (twice a week) with osteogenic induction medium consisting of 100 nM dexamethasone, 10 mM β-glycerophosphate, 0.2 mM ascorbate (all from Sigma-Aldrich, USA), and 10% FCS in DMEM/F-12 basal medium. Osteogenic differentiation was confirmed by the enhancement of alkaline phosphatase (AP) expression using histochemical staining, and the deposition of mineralised matrix by 0.2% alizarin red-S staining.

For adipogenic differentiation [4], the cells were induced by three cycles of induction/maintenance using adipogenic induction medium consisting of 1 mM dexamethasone, 0.5 mM 3-isobutyl-1-methyl-xanthine (IBMX), 10 μg/ml recombinant human insulin, 100 mM indomethacin (all from Sigma-Aldrich), and 10% FCS, and using adipogenic maintenance medium consisting solely of 10μg/ml recombinant human insulin and 10% FCS. After completing the three cycles of induction/maintenance, the induced cells were incubated for another 7 days in adipogenic maintenance medium. Adipogenic differentiation was confirmed by the formation of neutral lipid-vacuoles stainable with 0.18% oil Red-O for 5 min (Sigma-Aldrich).

For chondrogenic differentiation [4], the cells were cultured by a micromass culture. Therefore 2.5×10^5^ cells were centrifuged in a 15-ml polypropylene tube at 150×*g* to form a pellet. Without disturbing the pellet, the cells were cultured for 4 weeks in 0.5 ml of complete chondrogenic differentiation medium including 10 ng/ml TGF-β-3 (Peprotech). Cells were fed twice a week. After the culture period, cryosections were analyzed by collagen II (Sigma-Aldrich) immunohistochemistry staining following the manufacturer’s instructions.

Primary normal human dermal fibroblasts served as negative controls in all three differentiation studies. Each experiment was repeated in quintet.

**CLI operation**

Animals received anesthesia induction by 2% isoflurane in 100% oxygen at a flow rate of 1 L/min, and then underwent aseptic femoral arterial ligation in continuous flow of 2% isoflurane in 100% oxygen at a flow rate of 0.3 L/min. Through mini-incision, the proximal site (distal to the bifurcation of common iliac artery) and distal site (proximal to popliteal artery) of the left femoral artery, as well as the superficial circumflex iliac artery and epigastric artery, were occluded using double knots by 7-0 silk suture (Ethicon, Johnson & Johnson, USA). The same levels of arterial ligation were kept for consistency of the model. The segment of femoral artery between the two sites was transected. The right hindlimb was kept intact as a non-ischemic limb.

**Functional scoring**

Semiquantitative functional assessments of the ischemic hindlimb were consecutively performed in a blinded manner using a modified clinical scoring for ambulatory impairment (0=toe flexion, 1=foot flexion, 2=no dragging but no plantar flexion, 3=foot dragging), and ischemic damage (0=no change, 1=mild discoloration, 2=moderate/severe discoloration, 3=necrosis, 4=amputation) [5].

**Luciferase assays**

*In vitro* Fluc assays were performed with different amounts of mADSCs^Fluc+GFP+^ using our previous protocols [6,7,8]. Cells were lysed using 1×Passive Lysis Buffer (Promega, USA) at 4°C. For every sample, 20 μl of supernatant was added to 100 μl of Luciferase Assay Reagent (LARII, Promega) and luminosity in relative light units (RLU) was detected by a 20/20n luminometer within Luciferase Assay System (Promega). PBS with no mADSCs^Fluc+^ was used as control. For *ex vivo* luciferase assay, tissues were removed from sacrificed mice, homogenised in PBS containing a protease inhibitor cocktail (Roche Applied Science, USA) and lysed with PLB. After centrifugation at 14,000 rpm for 10 min at 4°C, the supernatant was collected. Luciferase activity was measured using Luciferase Assay System. All samples were conducted in quintet.

**Histological analysis of mADSCs-induced angiogenesis**

Immunohistochemistry analysis was performed to visualize CD31^+^ vessels. Mice were sacrificed on day1/day21. Left gastrocnemius tissues were removed and fixed in 4% paraformaldehyde at room temperature for 24 h. Paraffin-embedded tissue was cut into 5-μm thick sections. After deparaffinization, sections were sequentially incubated with 0.3% hydrogen peroxide for 10 min, blocking solution for 1 h at room temperature and rat monoclonal anti-CD31 (1:50, ab7388, abcam) overnight at 4°C. Horseradish peroxidase (HRP)-conjugated goat anti-rat was used as secondary antibody (1:200, A10549, Invitrogen). Sections were stained with diaminobenzidine (DAB) and counterstained with hematoxylin (R&D system, USA).

We further adopted immunofluorescence assay to show the relationship of mADSCs^Fluc+GFP+^ and CD31^+^ microvasculature. Frozen tissue was cut into 10-μm thick sections. The sections were subsequently stained with rat monoclonal anti-CD31 (1:100) overnight at 4°C, followed by the staining of Alexa Fluor-594 (AF-594) labeled rabbit anti-rat IgG (1:200, Invitrogen) and FITC-conjugated goat anti-GFP (1:200, ab6662, abcam) for 1 h at 37°C. Sections were further stained with 4’,6-diamidino-2-phenylindole (DAPI) for total nuclei. Sections were stained using isotype nonspecific isotype IgG as control. Finally, the samples were imaged using laser confocal microscope (FluoView-FV1000, Olympus, JAPAN).

**Western blot and ELISA**

Gastrocnemius tissues were harvested on day0, day3 and day7 for Western blot and ELISA using our previous protocols [9,10]. Protein lysates were run on 5–20% SDS-PAGE gels and transferred onto nitrocellulose (NC) membrane. Membranes were blocked with 5% milk in 1×TBS-Tween-20 buffer and incubated overnight at 4°C with primary antibodies (dilution 1:2000 for anti-Akt, 1:1000 for others, all from Cell Signaling Technology, USA), including anti-: vascular endothelial growth factor receptor-2 (VEGFR2, #2472), phospho-VEGFR2 (Tyr951, #4991), mammalian target of rapamycin (mTOR, #2983), phospho-mTOR (Ser2448, #2971), Akt (#2920), phospho-Akt (Ser473, #4051) and β-actin (#4967). Immunoreactivity was detected by sequential incubation with HRP-conjugated antibodies and enzymatic chemiluminescence (#7003). Integrated optical density (IOD) of immunoblot was quantified using QuantiOne imaging software (Bio-rad, USA). ELISA was performed using the Quantikine ELISA kit (R&D Systems) to determine the concentration of vascular endothelial growth factor (VEGF), basic fibroblast growth factor (bFGF), hepatocyte growth factor (HGF) and stromal cell derived factor-1α (SDF-1α) in samples using manufacturer’s instructions.

**References**

1. Guide for the Care and Use of Laboratory Animals. 8th edition. Washington (DC): National Academies Press (US); 2011.

2. Zuk PA, Zhu M, Mizuno H, Huang J, Futrell JW, et al. (2001) Multilineage cells from human adipose tissue: implications for cell-based therapies. Tissue Eng 7: 211-228.

3. Zuk PA, Zhu M, Ashjian P, De Ugarte DA, Huang JI, et al. (2002) Human adipose tissue is a source of multipotent stem cells. Mol Biol Cell 13: 4279-4295.

4. Kern S, Eichler H, Stoeve J, Kluter H, Bieback K (2006) Comparative analysis of mesenchymal stem cells from bone marrow, umbilical cord blood, or adipose tissue. Stem Cells 24: 1294-1301.

5. Rutherford RB, Baker JD, Ernst C, Johnston KW, Porter JM, et al. (1997) Recommended standards for reports dealing with lower extremity ischemia: revised version. J Vasc Surg 26: 517-538.

6. Cao F, Lin S, Xie X, Ray P, Patel M, et al. (2006) *In vivo* visualization of embryonic stem cell survival, proliferation, and migration after cardiac delivery. Circulation 113: 1005-1014.

7. Cao F, Li Z, Lee A, Liu Z, Chen K, et al. (2009) Noninvasive de novo imaging of human embryonic stem cell-derived teratoma formation. Cancer Res 69: 2709-2713.

8. Cao F, Wagner RA, Wilson KD, Xie X, Fu JD, et al. (2008) Transcriptional and functional profiling of human embryonic stem cell-derived cardiomyocytes. PLoS One 3: e3474.

9. Xie X, Cao F, Sheikh AY, Li Z, Connolly AJ, et al. (2007) Genetic modification of embryonic stem cells with VEGF enhances cell survival and improves cardiac function. Cloning Stem Cells 9: 549-563.

10. Sun D, Huang J, Zhang Z, Gao H, Li J, et al. (2012) Luteolin limits infarct size and improves cardiac function after myocardium ischemia/reperfusion injury in diabetic rats. PLoS One 7: e33491.
